# Analysis of evidence appraisals for interventional studies in family medicine using an informatics approach

**DOI:** 10.1017/S1463423619000264

**Published:** 2019-08-22

**Authors:** Alain Nathan Sahin, Andrew Goldstein, Chunhua Weng

**Affiliations:** 1 Department of Biomedical Informatics, Columbia University, New York, NY, USA; 2 Primary Care, Bellevue Hospital Center/New York University School of Medicine, New York, NY, USA

**Keywords:** clinical informatics, critical appraisal, family medicine, journal comments, peer review

## Abstract

This study reports the first assessment of published comments in the family medicine literature using structured codes, which produced commentary annotations that will be the foundation of a knowledge base of appraisals of family medicine trials. Evidence appraisal occurs in a variety of formats and serves to shed light on the quality of research. However, scientific discourse generally and evidence appraisal in particular has not itself been analyzed for insights. A search strategy was devised to identify all journal comments indexed in PubMed linked to controlled intervention studies published in a recent 15-year period in major family medicine journals. A previously developed structured representation in the form of a list of appraisal concepts was used to formally annotate and categorize the journal comments through an iterative process. Trends in family medicine evidence appraisal were then analyzed. A total of 93 comments on studies from five journals over 15 years were included in the analysis. Two thirds of extracted appraisals were negative criticisms. All appraisals of measurement instruments were negative (100%). The participants baseline characteristics, the author discussions, and the design of the interventions were also criticized (respectively 91.7%, 84.6% and 83.3% negative). In contrast, appraisals of the scientific basis of the studies were positive (81.8%). The categories with the most appraisals were, most generally, those focused on the study design, and most specifically, those focused on the scientific basis. This study provides a new data-driven approach to review scientific discourse regarding the strengths and limitations of research within academic family medicine. This methodology can potentially generalize to other medical domains. Structured appraisal data generated here will enable future clinical, scientific, and policy decision-making and broader meta-research in family medicine.

## Glossary

Appraisal: Any evaluation of a trial or its reporting, any independent interpretation of the results, or any implication-making by the author of the appraisal (including application of the intervention or future research steps).
Appraisal concept: Text fragment containing an appraisal (e.g. “this COPD trial only examined mortality, neglecting dyspnea”).
Category: The general heading under which a knowledge acquisition can be classified. Synonymous with axial code. For example, the knowledge acquisition “Outcome Set Incomplete” would be categorized under the category “Study Design”.
Field-level study: Study on a specific discipline (e.g. family medicine or nursing).
Knowledge acquisition: Label that allows the classification of appraisals in a more general fashion, regardless of the disease or of the intervention. Synonymous with open code. For the COPD trial example above, the knowledge acquisition would be “Outcome Set Incomplete”.
Knowledge representation: A representation of knowledge that facilitates its use. For example, the Dewey Decimal System or Medical Subject Headings (MeSH) could be considered knowledge representations.
Sub-category: Further division of category that allows better classification of a knowledge acquisition (e.g. “Study Design, Outcomes).

## Introduction

Evidence-based medicine aims to integrate the best available evidence in decision-making processes (Sackett, 1997). Evidence appraisal, defined as “critical appraisal of published clinical studies via evaluation and interpretation by informed stakeholders” (Goldstein et al., 2017), is an important means of assessing the quality of published clinical research. Subsequently, evidence appraisal allows understanding of the characteristics, strengths, and flaws of research and the planning of knowledge translation and future research.

However, evidence appraisal, albeit ubiquitously present in journal comments, blogs, journal clubs and conference proceedings, is currently under-studied and appraisal knowledge, under-utilized. As part of an informatics research agenda (Goldstein et al., 2017; Sahin et al., 2018) on evidence appraisal, we have acquired appraisal data and have developed an initial evidence appraisal knowledge representation through an iterative terminology development process. This knowledge representation is a list of codes that can be used to classify appraisals. Other coding schemes or controlled vocabularies, such as Medical Subject Headings (MeSH), facilitate information retrieval and contextualization (Minguet *et al.*, 2014). Similarly, this knowledge representation for appraisal concepts seek to improve data management by enabling structured annotation and large-scale reasoning, which in the long-term will impart actionable knowledge for clinical, scientific, policy decision-making, and for meta-research. To our knowledge, this knowledge representation-based approach is the first of its kind in the evidence appraisal literature.

By using this approach in the context of family medicine research, we can tailor the knowledge representation to field-level needs and also have a better understanding of the field’s research in terms of its impacts and of its limitations. Previous field-level studies analyzed papers published in family medicine journals (Silagy *et al.*, 1994; Merenstein *et al.*, 2003; Pathman *et al.*, 2008; Jeon *et al.*, 2014). However, these analyses either focused on a small sample of old studies published more than 15 years ago, or only described the type of studies in journals without in-depth comprehensive appraisal of the evidence in these studies. Furthermore, no prior studies performed analyses that generated reusable, computable annotations, nor harnessed existing published evidence appraisals. Thus, the purpose of this study was to bridge this knowledge gap and to apply a new representation for annotating evidence appraisals to journal comments on controlled intervention studies in family medicine published in the past 15 years. On this basis, we draw conclusions to address the need for an assessment of the current state of family medicine research.

## Methods

**Figure 1.**
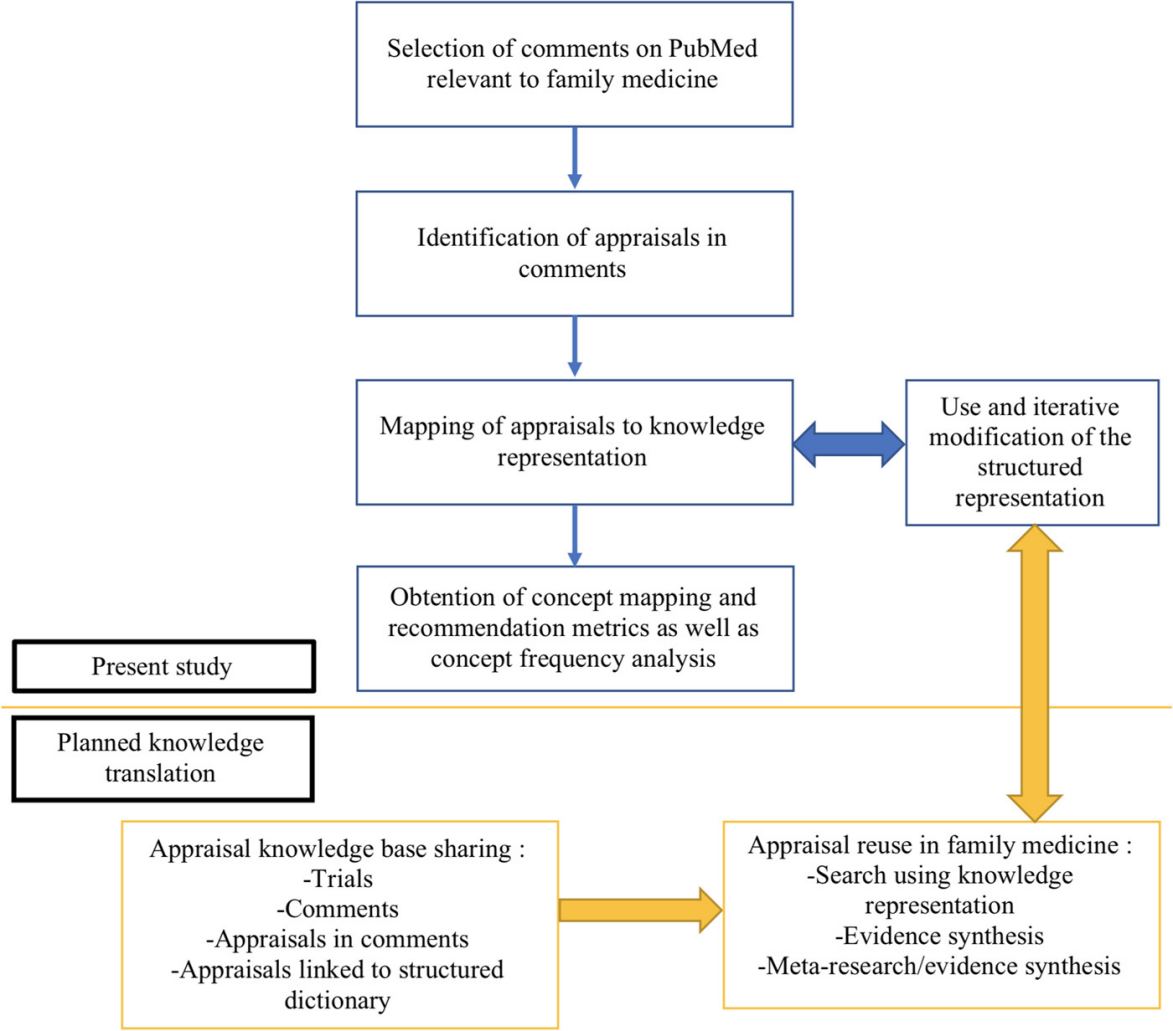
Workflow for harnessing, analyzing, and using evidence appraisals

This article does not contain any research with human participants or animals performed by any of the authors. Ethics approval is not required for this type of review. Figure 1 illustrates our workflow framework for data extraction, annotation, analysis and use.

### Step 1: study and comment selection

The selection process was comprised of two steps. First, to identify controlled intervention studies published in the last 15 years, we used the National Library of Medicine’s PubMed database. The PubMed database was searched between October 2002 and December 2017 for clinical trials in major family medicine journals which had published journal comments linked to them. The search criteria (Appendix A) were devised by taking into account the five eligible journals with the highest number of total cites on Thomson’s Journal Citation Reports (JCR). *American Family Physician* was excluded from the search criteria as it does not publish controlled intervention studies. Only controlled intervention studies, with the presence of an intervention arm and at least one comparison arm, were kept. Randomization was not an exclusion criterion.

Second, all entries in PubMed having a comment on the studies satisfying the search criteria in the first step were extracted and evaluated for eligibility. An eligible comment included a letter to the editor, an editorial commentary, or other comment directly discussing published intervention trials with comparators. We refer to these as “comments” even if they are described as letters or editorials. Exclusion criteria were entries in PubMed that merely cited the study or entries that were author replies.

### Step 2: appraisal concept acquisition and annotation process

Appraisal concepts were then collected by the first author (Author 1), a trained medical student in his third year of medical studies. After reading the abstract of the original study, the full article if need be, and the comment, specific appraisal text fragments were collected. These fragments which occur mid-sentence and mid-paragraph, were the shortest possible fragments discussing a specific appraisal. Then, each text fragment was mapped to a structured term (i.e., code) expressing the appraisal concept using an original knowledge representation.

If the representation did not have a fitting concept to the text fragment, a new concept was proposed. New concepts were then validated by the second author (Author 2) who had initially developed the original knowledge representation. The second author was, at the time, a Clinical Instructor in Medicine with a Master’s of Public Health and a postdoctoral research fellow in bioinformatics. To arrive at more precise structured terms and at relevant term categories the representation was further refined through an iterative process using the open coding method first and then the axial coding method (Saldaña, 2016; Meng *et al.*, 2017).

Open coding consists of identifying appraisal concepts that can be applied in a general fashion, regardless of disease or intervention being studied (e.g. “Inapplicability Due to Treatment Infeasibility”) (Saldaña, 2016; Samuel *et al.*, 2017). Axial coding then classified these concepts into overarching categories (e.g. “Study Design”) (Saldaña, 2016; Samuel *et al.*, 2017). In this study, the informatics term “knowledge acquisition” (Huang *et al.*, 2012) refers to manual review of journal comments, identification of text fragments where the comment author is expressing an appraisal, and open coding of this appraisal. Axial codes are termed categories. These categories can be subdivided using subcategories (e.g. “Study Design, Intervention”) indicating that the Study Design category has an Intervention subcategory.

### Step 3: concept mapping and recommendation metrics

The manual extraction and categorization of the appraisal concepts allowed for analysis of trends in family medicine research. We provided quantification and lists of newly acquired knowledge (e.g. “Inapplicability Due to Treatment Infeasibility”), the most common appraisal categories (e.g. Study Design (all)), and subcategories (e.g. Study Design, Intervention).

## Results

**Figure 2.**
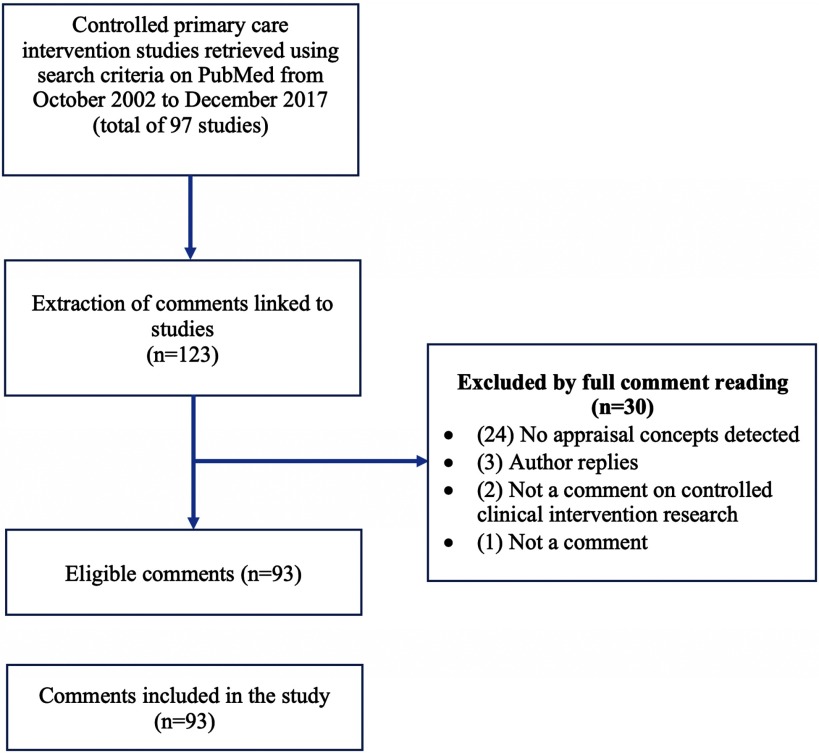
Flow chart of search results and eligibility screening

Figure 2 presents the results of the search and eligibility screening. A total of 93 comments on studies published in five journals between October 2002 and December 2017 were included in our analysis. By adapting our structured knowledge representation to the field of family medicine, our tool grew to contain a total of 20 categories, 39 subcategories, and 398 knowledge acquisition codes.

After appraisal concept identification, we separated the concepts into three groups (positive connotation, neutral connotation, negative connotation) depending on whether the appraisal suggested a strength of the study, a remark independent of the control of the authors (e.g. Future Research Should Help Find Groups Who Will Most Benefit from Intervention), or an area of desired improvement. About two thirds (67%) of the appraisals were negative. Neutral comments and positive comments comprised respectively 4% and 29% of appraisals.

The most common high-level appraisal categories and their negativity are shown in Table 1. We see that “Study Design” is the one that is the most prominent with over twice the number of entries than the second most common “Trial Reporting” or the third most common “Results”. Table 2 presents the most common appraisal categories with their three subcategories. “Scientific Basis”, “Knowledge Translation”, and “Study Design, Intervention” are the ones most present in the journal comments.

**Table 1.** Most common appraisal categories and their negativity

Categories	Rank	Total	Positive Comments	Neutral Comments	Negative Comments	Percent Negative (%)
Study Design (all)	1	83	22	1	60	72.3
Trial Reporting (all)	2	39	6	0	33	84.6
Results (all)	3	36	10	0	26	72.2
Scientific Basis	4	33	27	0	6	18.2
Knowledge Translation	5	28	7	2	19	67.9
Future Research Implications	6	17	1	8	8	47.1

**Table 2.** Most common appraisal subcategories and negativity

Subcategories	Rank	Total	Positive Comments	Neutral Comments	Negative Comments	Percent Negative (%)
Scientific Basis	1	33	27	0	6	18.2
Knowledge Translation	2	28	7	2	19	67.9
Study Design, Intervention	3	18	3	0	15	83.3
Future Research Implications	4	17	1	8	8	47.1
Results, Outcomes	5	13	9	0	4	30.8
Trial Reporting, Discussion	5	13	2	0	11	84.6

Table 3 ranks the most common subcategories in terms of the proportion of negative comments in each category. The comments on measurement instruments used in recent family medicine studies were all negative. Similarly, the participant baselines, the trial reporting with regards to the discussion of the scientific papers, and the study design of the interventions and outcomes were mostly negatively criticized. Nevertheless, the scientific basis of the articles received a majority of positive comments. Notably, these positive appraisals on the scientific basis may be genuinely stated, but are commonly used to start journal comments and may be a polite way of prefacing negative appraisals.

**Table 3.** Ten most common appraisal subcategories ranked by negativity

Subcategories	Total	Positive Comments	Neutral Comments	Negative Comments	Percent Negative (%)
Study Design, Measurement Instrument	12	0	0	12	100
Results, Participants Baseline	12	1	0	11	91.7
Trial Reporting, Discussion	13	2	0	11	84.6
Study Design, Intervention	18	3	0	15	83.3
Study Design, Outcomes	11	2	0	9	81.8
Study Design, Power	11	3	0	8	72.7
Study Design, Study Arms	10	3	0	7	70
Knowledge Translation	28	7	2	19	67.9
Future Research Implications	17	1	8	8	47.1
Results, Outcomes	13	9	0	4	30.8
Scientific Basis	33	27	0	6	18.2

Table 4 lists the most common appraisal knowledge acquired from the journal comments. As these appraisal knowledge acquisitions were numerous, they tend to polarize the connotations (positive or negative) of their corresponding subcategory. We remarked that “Study Inadequately Powered” was the 6th most common knowledge acquisition. We also observed that one randomized controlled trial (RCT) study was criticized for using an ‘as-treated’ analysis.

**Table 4.** Most common appraisals

Frequent Appraisals	Corresponding Subcategory	Rank	Total
Addresses Prior Scientific Concern	Scientific Basis	1	22
Author Interpretation Inconsistent with Results	Trial Reporting, Discussion	2	9
Lacks Applicability Based on Dissimilarity to Recruited Participants	Results, Participants Baseline	3	8
Outcome Set Incomplete	Study Design, Outcomes	3	8
Study Interventions Inadequately Reported	Trial Reporting, Study Design, Interventions	3	8

## Discussion

In this study, we have adopted and expanded an evidence appraisal knowledge representation to assess recent studies published in major family medicine journals via the analysis of linked journal comments. The content of all eligible comments on studies of the past 15 years were fragmented and classified. We have combined different tools to highlight recent challenges and accomplishments of family medicine. Our main finding was that the field of family medicine produces studies with relevance to clinical practice but needs to address study design difficulties.

Our findings showed that journal comments were inclined to highlight the weaknesses of studies. As journal comments tend to be published when they add intellectual content to the subject at hand, it is possible that evidence appraisal through journal comments could mainly be used to highlight flaws of studies. Our research here enables further necessary appraisal validation work that is required to determine if this negativity is a bias or if it is appropriate. The use of our representation in other analyses would allow for comparison and show if family medicine studies tend to receive a larger proportion of negative comments than in other fields. Likewise, the qualitative nature of some family medicine research could partly explain the predominantly negative comments on the measurement instruments. Involving stakeholders in study design, such as in participatory research, could improve the studies and reduce negative post-publication appraisals (Cargo and Mercer, 2008). Of note, in 2010, Delaney, in her last editorial as Editor in Chief of *Family Practice*, suggested more training in informatics as a way to ameliorate the academic discipline of family medicine and overcome present study barriers (Delaney, 2010).

The relevance of studies published in family medicine remains high as comment authors perceive the studies to fill important gaps in the literature. Silagy *et al.* reported similar findings in their article on older studies (Silagy *et al.*, 1994). We also showed that many comments then report on knowledge translation and future research implications, as these will lead to clinical impact.

Our findings also potentially demonstrate progress in family medicine research rigor. Silagy *et al.* also reported on their analysis of 55 RCTs that many trials did not use an ‘intention-to-treat’ analysis (Silagy *et al.*, 1994); in our study, only a single trial was criticized for using an ‘as-treated’ analysis. In addition, Silagy *et al.* showed many studies lacked mention of whether the RCT had ethics approval as this was a new requirement at the time (Silagy *et al.*, 1994); no comment identified such a lack in the studies published recently. Other issues still persist: Silagy *et al.* described several studies failing to include power calculations and being underpowered. In our assessment, “Study Inadequately Powered” was the 6^th^ most common knowledge acquisition. As recent studies (Sahin *et al.*, 2014; Robitaille *et al.*, 2014) have shown, both difficulties in patient and physician recruitment are important barriers to clinical practice research that must be overcome.

Our study shows that the most common category was “Study Design”. In the past, Merenstein *et al.* concluded that there was a lack of family medicine studies published in the year 2000 with high-quality designs (Merenstein *et al.*, 2003). This challenge is potentially related to the unique characteristics of patients and physicians involved in family medicine (Bower *et al.*, 2009), as well as what was termed “the dispersed nature of the primary care setting” (Bower *et al.*, 2009). Our review identified several journal comments remarking that the applicability of the studies to populations was affected by recruited patient dissimilarities.

Patient care is at the center of family medicine. Nevertheless, Merenstein *et al.* noted the need for more patient-oriented clinical research (Merenstein *et al.*, 2003). In the same vein, our study showed that the third most common knowledge acquisition was “Outcome Set Incomplete” indicating that key patient-oriented outcome measures need broader adoption in family medicine clinical research. Isolated, rarer, issues directly related to patient-centered care were also noted with our classification (e.g. “Introduction Lacks Appropriate Discussion of Existing Evidence of Intervention Harms”). Conversely, literature shows that there is a desire to innovate for more and better patient-centered research, with models of patient partnerships (Karazivan *et al.*, 2015; Pomey *et al.*, 2015) and national collaborations (James *et al.*, 2013) being created in the last years.

Of note, at the field-level, scientific discourse on family medicine trials is minimal. We identified 93 comments appraising trials in 5 journals over 15 years, indicating 1.2 comments on trials per year per journal. The research enterprise, journals, and the family medicine field could foster more vibrant published scientific discourse to further enhance research quality.

There are limitations to our study. Our study only analyzed a single source of evidence appraisals, published journal comments listed on PubMed. This source is not definitive as many trials are worthy of comments, and many appraisals exist beyond journal comments, including documented unpublished ones (e.g. social media commentary) and undocumented ones (e.g. journal club discourse). Furthermore, those comments needed to be linked to trials published in the journals we specified. Several studies published by family medicine researchers are published in a wide variety of journals, both in terms of discipline and impact factor. In addition, our analysis of the comments primarily reflects what the authors of those comments wished to put emphasis on. Thus, comments do not need to systematically and comprehensively critically appraise a study. With regards to the methodology we have developed, our representation is early stage and being developed in an iterative process. This requires long-term harnessing of appraisal data with continual updating and reframing of entries, just as diabetes mellitus was later reframed as Type 1 and Type 2. With a variety of different text fragments in different contexts, our representation entries will be further validated and modified, just like any controlled vocabulary applied in dynamic settings. Future validation work will include this, as well as annotation-derived development which additionally yield documentation for all terms to ensure validity and reproducibility. Nevertheless, all newly acquired knowledge found in our review was validated by two reviewer consensus.

This study is a comprehensive assessment of controlled intervention appraisals available on PubMed in the discipline of family medicine in the past 15 years. Our results provide a snapshot of the strengths and the obstacles that academic family medicine faces. Although the field rapidly advances, some issues, mainly related to designing studies, remain, though as others have noted, the areas of needed improvement are not unique to family medicine (Merenstein *et al.*, 2003). Our representation will ultimately also allow for the possibility of comparing the main challenges of fields, and the annotations this research has yielded will be the foundation of a knowledge base enabling broader meta-research.

## Supplementary material

For supplementary material accompanying this paper visit https://doi.org/10.1017/S1463423619000264. click here to view supplementary material
