# Supplementary material for: Analysis of evidence appraisals for interventional studies in family medicine using an informatics approach
Source: Prim Health Care Res Dev. 2019 Aug 22;20:e123. doi: 10.1017/S1463423619000264 (PMC6713885; doi:10.1017/S1463423619000264)
Supplement: Supplementary file 1 [file S1463423619000264sup001.docx]

**Appendix A : Search Strategy**

The following search strategy was developed for PubMed :

(("J Am Board Fam Pract"[journal] or "J Am Board Fam med"[journal] or "ann fam med"[journal] or "fam pract"[journal] or "BMC fam pract"[journal] or "Br J Gen Pract"[journal]) hascommentin "clinical trial"[pt])

Last retrieval by Author 1 on December 27, 2017.

**Figure 1 Workflow for harnessing, analyzing, and using evidence appraisals**

**Figure 2 Flow chart of search results and eligibility screening**
